# Response to 5-Fluorouracil-Based Chemotherapy in a Patient with Metastatic Colonic-Type Adenocarcinoma Arising in a Primary Mediastinal Teratoma

**DOI:** 10.1155/2012/729278

**Published:** 2012-09-23

**Authors:** Arunee Dechaphunkul, Gilbert Bigras, Michael Sawyer

**Affiliations:** ^1^Department of Oncology, Faculty of Medicine and Dentisty and Cross Cancer Institute, University of Alberta, Edmonton, AB, Canada T6G 1Z2; ^2^Holistic Center for Cancer Study and Care (HOCC-PSU) and Division of Medical Oncology, Department of Internal Medicine, Faculty of Medicine, Prince of Songkla University, Songkhla 90110, Thailand; ^3^Department of Laboratory Medicine and Pathology and Cross Cancer Institute, University of Alberta, Edmonton, AB, Canada T6G 1Z2

## Abstract

Germ cell tumor with somatic malignant transformation is an uncommon phenomenon occurring about 7% of all mediastinal teratomas. Among all transformed component, sarcoma appears to be the most frequent histology, followed by primitive neuroectodermal tumor (PNET) and adenocarcinoma. To our knowledge, there were 3 cases of colonic-type adenocarcinoma arising in a primary teratoma have been reported to date. However, none of them received chemotherapy directed to transformed histology given localized disease at presentation. We, therefore, report here the first case of patient who achieved good response from chemotherapy directed to transformed histology, which confirms the importance of chemotherapy regimen used.

## 1. Introduction

Germ cell tumors (GCTs) are the most common solid tumor in men between the ages of 15 and 35 years. Although majority of GCTs originate from the gonads, 1–5% of GCTs arise from extragonadal sites. Extragonadal GCTs typically arise in the midline location, and specific sites vary with age. In adults, the most common sites, in order of frequency, are mediastinum, retroperitoneum, and pineal gland and suprasellar regions [1–3]. GCTs account for approximately 15% of mediastinal tumors in adult. Mediastinal GCTs also demonstrate a wide spectrum of histological classification proposed by World Health Organization (WHO) [4]. Among these, teratoma is the most common type with the frequency of 44% [5]. Occasionally, GCTs develop a nongerm cell or somatic component. When patient was diagnosed with GCTs with somatic malignant transformation, the important question is which chemotherapy regimen should be used: regimens specific to GCTs or transformed histology? Here, we report a case of patient with mediastinal GCTs with somatic malignant transformation who achieved good response to chemotherapy directed to transformed histology. 

## 2. Case Report 

**Figure 1:**
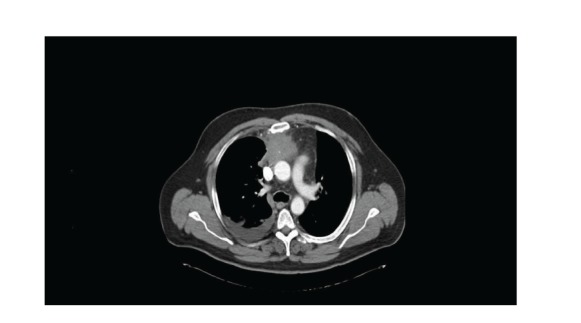


**Figure 2:**
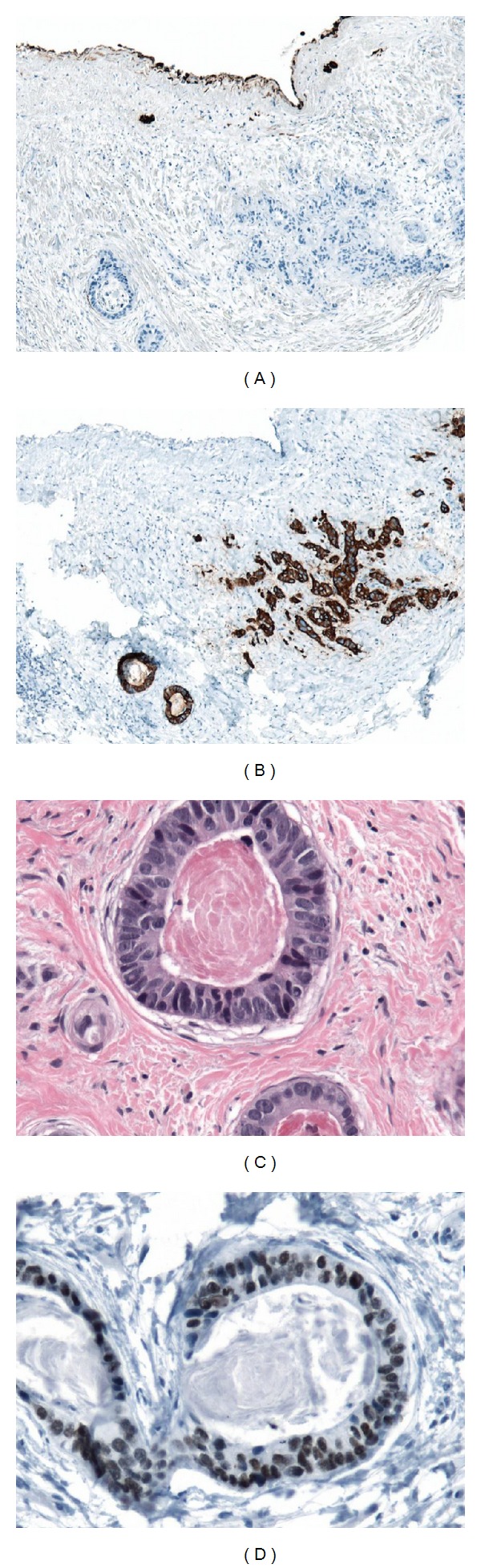
Four pictures from a pleural biopsy showing metastatic adenocarcinoma. (a) Cytokeratin 7 antibody: malignant epithelial structures are negative while benign mesothelial lining is positive (magnification 100x). (b) Cytokeratin 20 antibody: malignant epithelial structures are positive while benign mesothelial lining is negative. Malignancy shows well (left) and poor (right) differentiation (magnification 100x). (c) picture of the well (glandular) differentiation. Malignant epithelial cells are columnar (H&E staining, magnification 400x). (d) CDX-2 antibody: moderate to strong nuclear staining (magnification 400x).

A 47-year-old male was referred to our hospital with a 2-month history of chest tightness and shortness of breath. He denied fever, cough, or other constitutional symptoms. His medical history included well-controlled hypertension for 10 years. Physical examination revealed two firm 1 cm right supraclavicular lymph nodes. Respiratory examination showed decreased breath sounds and dullness on percussion of the right lung base. Computed tomography (CT) scan of the chest and abdomen demonstrated a 6 cm anterior mediastinal mass with a few punctate areas of calcification and right pleural effusion (Figure 1). There was no evidence of predominant pulmonary nodule to suggest a primary bronchogenic cancer or any intra-abdominal abnormalities. Serum tumor markers showed slight elevation of alpha-fetoprotein (AFP) at 15 *μ*g/L (normal < 9 *μ*g/L), normal *β*-human chorionic gonadotropin (*β*-hCG) < 5 U/L (normal < 5 U/L), and lactate dehydrogenase (LDH) 116 U/L (normal 100–225 U/L). Right thoracentesis with talc pleurodesis was performed to alleviate symptoms. Pleural fluid cytology revealed benign and reactive mesothelial cells and occasional groups of atypical cells, but was not diagnostic. Pathology from the right pleura revealed well-formed malignant gland compatible with a well-differentiated adenocarcinoma. Further immunohistochemical (IHC) analysis was strongly positive for cytokeratin (CK) 20 and CDX2, but negative for CK7, [thyroid-transcription factor-1] (TTF-1), AFP, *β*-hCG, PLAP, CD30, and OCT3/4 (Figures 2(a)–2(d)). In addition, CT-guided biopsy of the anterior mediastinal mass revealed moderately differentiated adenocarcinoma with no morphological evidence of classical thymoma or germ cell tumor. Identical IHC findings were shown from both pleura and anterior mediastinal mass tissues. On the basis of IHC profile in conjunction with morphology, this was consistent with colonic-type adenocarcinoma. The patient went on to have colonoscopy which failed to show any primary colorectal cancer. Therefore, the most probable diagnosis in this case was mediastinal germ cell tumor with somatic malignant transformation, colonic-type adenocarcinoma arising in a teratoma. He was treated with 5-fluorouracil-(5FU-) based chemotherapy (5FU/leucovorin/irinotecan—FOLFIRI) directed to the transformed histology. After 3 months of therapy, he achieved clinical response and radiologically stable disease. He received a total 7 cycles of FOLFIRI chemotherapy, but unfortunately passed away from a stroke with 9-month overall survival. 

## 3. Discussion

Primary mediastinal germ cell tumor (PMGCT) comprises approximately 10% of all mediastinal tumors [5]. Among all surgically resected PMGCT, mature teratoma accounts for 80% of cases [6]. The prognosis in patients with pure mature teratoma is generally excellent with a complete surgical resection. The well-known association between mediastinal nonseminomatous germ cell tumors (GCTs) and hematological neoplasia has been reported with nearly 50 cases published to date [5], in which patients have an extremely poor prognosis with a median overall survival of only 5 months after diagnosis [7]. Apart from the hematological neoplasia, the teratomatous element can also transform into various somatic malignancies [5].

GCTs with somatic malignant transformation (SMT) is defined as a GCT accompanied with a somatic-type malignant counterpart. It is an infrequent phenomenon occurring about 7% of all mediastinal teratomas [8]. The site of SMT may be at the primary site, metastatic sites, or both. Teratoma with SMT can be divided in two clinical conditions: (1) radiation- or chemotherapy-induced SMT or (2) *de novo* SMT [9]. Among all transformed component, sarcoma especially rhabdomyosarcoma appears to be the most frequent histology, followed by primitive neuroectodermal tumor (PNET) and adenocarcinoma [5, 8]. Previous studies reported adenocarcinoma would generally occur later in comparison to other types of malignancies (a few years after a diagnosis of GCTs) [10, 11]; however, our patient was diagnosed with PMGCT and SMT simultaneously. 

Teratoma with SMT is considered a chemoresistant tumor, therefore surgery is the mainstay treatment, particularly for patients with localized disease. Patients who underwent complete resection of the malignant component appear to have an excellent prognosis [12], whereas for patients with metastasis, which represents a majority of patients, the prognosis is generally poor with median overall survival of only 9 months [13]. In this advanced setting, chemotherapy should be directed to transformed histology especially in patients with single cell type. However, local therapy after chemotherapy is still an important component to achieve maximum response [11]. Although histology of the somatic malignancy does not have a major impact on prognosis, adenocarcinoma usually behave aggressively with higher resistance to therapy [9, 10]. 

Colonic-type adenocarcinoma arising in a mature teratoma has been reported with 3 cases published to date. Chu et al. described a young female with diagnosis of primary retroperitoneal teratoma with adenocarcinomatous transformation predominantly composed of signet ring cell carcinoma and intestinal-type adenocarcinoma who developed distant metastasis only 2 months after surgery despite receiving adjuvant radiotherapy and cisplatin-based combination chemotherapy [14]. Cheung and Cao reported a case of 47-year-old female who remained disease-free for 18 months at the time report after undergoing complete surgical excision for her colonic-type adenocarcinoma arising in a primary retroperitoneal mature cystic teratoma [15]. This confirmed the importance of complete surgical resection in patients with localized disease. Most recently, Khurana et al. reported a young male who have been symptom-free for the past 6 months after complete surgery for his colonic-type adenocarcinoma arising in a mediastinal mature cystic teratoma [13]. 

In conclusion, our patient is the first case, to our knowledge, of a metastatic colonic-type adenocarcinoma arising in a teratoma who achieved response from 5-fluorouracil-based chemotherapy directed to the transformed histology. This affirms the importance of chemotherapy regimen used. Unfortunately, he passed away from a noncancer-related cause.
